# LPS induces IL-10 production by human alveolar macrophages via MAPKinases- and Sp1-dependent mechanisms

**DOI:** 10.1186/1465-9921-8-71

**Published:** 2007-10-04

**Authors:** Hugues Chanteux, Amélie C Guisset, Charles Pilette, Yves Sibille

**Affiliations:** 1 Unit of microbiology, Université Catholique de Louvain, Av Hippocrate 54, B-1200 Brussels, Belgium

## Abstract

**Background:**

IL-10 is a cytokine mainly produced by macrophages that plays key roles in tolerance to inhaled antigens and in lung homeostasis. Its regulation in alveolar macrophages (HAM), the resident lung phagocytes, remains however unknown.

**Methods:**

The present study investigated the role of intracellular signalling and transcription factors controlling the production of IL-10 in LPS-activated HAM from normal nonsmoking volunteers.

**Results:**

LPS (1–1000 pg/ml) induced *in vitro *IL-10 production by HAM, both at mRNA and protein levels. LPS also activated the phosphorylation of ERK, p38 and JNK MAPkinases (immunoblots) and Sp-1 nuclear activity (EMSA). Selective inhibitors of MAPKinases (respectively PD98059, SB203580 and SP600125) and of Sp-1 signaling (mithramycin) decreased IL-10 expression in HAM. In addition, whilst not affecting IL-10 mRNA degradation, the three MAPKinase inhibitors completely abolished Sp-1 activation by LPS in HAM.

**Conclusion:**

These results demonstrate for the first time that expression of IL-10 in lung macrophages stimulated by LPS depends on the concomitant activation of ERK, p38 and JNK MAPKinases, which control downstream signalling to Sp-1 transcription factor. This study further points to Sp-1 as a key signalling pathway for IL-10 expression in the lung.

## Background

Strategically located on the alveolar surface, alveolar macrophages represent highly specialized macrophages that function primarily in lung defence against inhaled particle matter, microorganisms and environmental toxins. Among microorganisms, gram-negative bacteria and more precisely, the lipopolysaccharide (LPS) component of the outer cell wall, is a very potent activator of macrophages. LPS binds to LPS-binding protein and is delivered to the cell surface receptor CD14, before being transferred to the transmembrane signaling receptor toll-like receptor 4 (TLR4) and its accessory protein MD2 [1]. LPS stimulation activates several intracellular signaling pathways including the three mitogen-activated protein kinase (MAPK) pathways: extracellular signal-regulated kinases (ERK) 1 and 2, c-Jun N-terminal kinase (JNK) and p38. These signalling pathways in turn activate a variety of transcription factors which coordinate the induction of many genes encoding inflammatory mediators as well as anti-inflammatory cytokines.

The control of inflammatory responses is critical to the host to allow resolution and avoid tissue damage. IL-10 is a key anti-inflammatory factor and pleiotropic cytokine produced by a variety of cell types among which monocytes/macrophages are the main sources [2]. IL-10 mediates the inhibition of pro-inflammatory cytokines such as TNF-α, IL-8, IL-6, IL-1β, IL-12 [3-7]. IL-10 has also been shown to inhibit antigen-presenting cell function, including the maturation of dendritic cells [8] and the expression of MHC class II and co-stimulatory molecules [9,10]. IL-10 gene regulation can occur both at the transcriptional and posttranscriptional levels [11]. Several studies have shown that the transcription factor Sp1 plays an important role in IL-10 transcription (an Sp1 responsive element in the IL-10 promoter is localized at -89 to -78) [12-14]. Moreover, detailed studies showed that p38 mitogen-activated protein regulates LPS-induced activation of Sp1 in THP-1, a human monocytic cell line [14]. The STAT3 transcription factor may also bind to an element in the IL-10 promoter gene and the use of a dominant negative form of STAT3 was able to decrease IL-10 transcription [15]. More recently, the protooncogene c-Maf has been shown to be an essential transcription factor for IL-10 gene expression in macrophages [16] while a role for C/EBP in cooperation with Sp1 has also been suggested [17]. However, the intracellular signalling pathways governing IL-10 gene regulation in human alveolar macrophages are poorly understood. Thus, alveolar macrophages are the main source of IL-10 in the alveoli where they play an important role to control lung homeostasis. One study on human alveolar macrophages [18] showed that activation of PKC decreases IL-10 production whereas activation of protein phosphatases PP1 and PP2A enhance IL-10 secretion. In the present work, we evaluate the ability of human alveolar macrophages to produce IL-10 upon LPS stimulation and the role of MAPkinases (ERK, p38 and JNK) and Sp1 transcription factor as intracellular signals leading to IL-10 expression.

## Methods

### Reagents

LPS from *Salmonella typhimurium*, PMSF, Nonidet, DTT, BSA, Tween 20, Thiazolyl Blue Tetrazolium Bromide and Actinomycin D were purchased from Sigma (Sigma Chemical Co., St Louis, MO). PD98059, SB203580 were purchased from BioMol (Plymouth Meeting, PA) and SP600125 from AG Scientific (San Diego, CA). Anti-CD14 was purchased from R&D Systems (Abingdon, UK). All other reagents were from VWR International (Darmstadt, Germany).

### Isolation of Human Alveolar Macrophages (HAM)

HAM were obtained from bronchoalveolar lavages from normal non smoking volunteers as previously described [19]. Briefly, the lavage fluid was passed through a layer of sterile gauze to remove gross mucus and then centrifuged at 500 *g *for 10 min at 4°C to separate cells from fluid. The cell pellet was washed twice in complete culture medium : RPMI 1640 medium (Cambrex Corporation, East Rutherford, NJ) supplemented with 10% decomplemented (30 min at 56°C) FCS, 2 mM L-glutamine, 100 U/ml penicillin and 100 μg/ml streptomycin. HAM were >95% pure with less than 1% of neutrophils and monocytes. HAM were allowed to adhere during 30 min and non-adherent cells were removed by two washes.

### Nuclear extract and Electrophoretic Mobility Shif Assay (EMSA)

Nuclear extracts were prepared from HAM as described by Carter with minor modifications as reported previously [19]. 1.10^6 ^HAM were washed in cold PBS and collected in 400 μl of ice-cold EMSA lysis buffer (10 mM HEPES pH 7.9, 10 mM KCl, 2 mM MgCl_2_, 2 mM EDTA, 1 mM DTT, 1 mM PMSF) supplemented with 10 μg/ml of each protease inhibitors (leupeptin, aprotinin, pepstatin, trypsin inhibitor) and then incubated on ice for 10 min. Nonidet (NP-40) 10% was added to lyse the cells which were vortexed and centrifuged 1 min at 4°C at 13000 rpm. Nuclei were resuspended in 25 μl of extraction buffer (50 mM Hepes pH 7.9, 10% glycerol, 50 mM KCl, 300 mM NaCl, 0,1 mM EDTA, 1 mM DTT, 0.5 mM phenylmethylsulfonylfluoride and protease inhibitors) for 30 min on ice. The nuclear suspension was then centrifuged 3 min at 13000 rpm and supernatant stored at -70°C until use. Proteins were assayed using the Blue coomassie method. Sp1 consensus oligonucleotides (5'-ATTCGATCG**GGGCGG**GGCGAGC-3') were purchased from Invitrogen Life Technologies (Carlsbad, CA) and were 3' biotinylated using Biotin 3' End DNA Labeling Kit (Pierce, Rockford, IL) following manufacturer's instructions. EMSA was performed using the LightShift Chemiluminescent EMSA kit (Pierce, Rockford, IL). Briefly, 2, 5 μg of nuclear extract was incubated at room temperature during 20 min with 20 fmol of biotinylated double-stranded oligonucleotide in presence of 50 ng of salmon sperm and reaction buffer. The protein-DNA complexes were separated on a 7% non-denaturating polyacrylamide gel and bands were visualized using Biorad Chemidocs XRS apparatus (Bio-Rad Laboratories, Hercules, CA).

### RNA extraction and real-time PCR

Total RNA was extracted from 10^6 ^HAM using TRIzol reagent (Invitrogen) and 2 μg of total RNA was used to generate first strand cDNA synthesis using Superscript II (Invitrogen/Life Technologies, Carlsbad, CA). The reaction mix containing 1 μg of RNA, poly-dT, and 10 mM dNTP mix was diluted to 24 μl in sterile water, heated to 65°C for 5 min, and chilled on ice for 1 min. First strand synthesis was then performed in 50 μl total reaction volume by adding 50 mM Tris (pH 8.3), 75 mM KCl, 3 mM MgCl_2_, 20 mM DTT, 40 U of RNaseout, and 200 U of Superscript II reverse-transcriptase enzyme (Invitrogen) at 42°C for 1 h. The reaction was inactivated by heating at 72°C for 10 min. cDNA was stored at -20°C until amplification. The quantitative PCR was performed by real-time PCR on a Lightcycler System (Roche Applied Science, Basel, Switzerland) using predevelopped primers set for human IL-10 (Search LC, GmbH Heidelberg, Germany). PCR conditions were those described in manufacturer's instruction. The reaction mix contains 5 μl cDNA, 1 mM of primers, water and Master mix (Faststart DNA Master plus Sybr Green I-Roche Applied Science) in a final volume of 20 μl. β-actin primers were designed following sequence published in GenBank (access number : M10277) in two different exons. They were synthesized by Life technologies : sense : 5'-gtgacattaaggagaagctgtgcta-3' (position : 2294–2317), antisense : 5'-cttcatgatggagttgaaggtagtt-3' (position : 2588–2612). PCR conditions were : denaturation at 95°C, 10 s – hybridization, 60°C, 5 s – elongation, 72°C, 7 s. After amplification step, a melting curve is performed to ensure that only one product has been amplified. Moreover, separation of the products on 2% agarose gel confirmed the size of the amplicon.

### IL-10 ELISA

IL-10 was assayed in the supernatant by ELISA using a pair of antibodies (BD Biosciences, San Diego, CA) following manufacturer's instructions. The sensitivity of the ELISA was 1.5 pg/ml.

### Western-Blot analysis

5 × 10^5 ^HAM were collected in 200 μl Laemmli sample buffer and heated at 100°C, 5 min to denature proteins. 20 μl of protein lysate was loaded onto a 12% SDS-PAGE gel and run at 180 V for 1 h. Cell proteins were then transferred to nitrocellulose (Hybond-C, Amersham Biosciences, UK) membrane at 70 mA for 1 h 30 at room temperature. Membrane was blocked with 5% BSA in TTBS (Tris-Buffered saline with 0.1% Tween 20) for 1 h at room temperature, washed, and then incubated with the primary Ab 1/1000(anti-phospho ERK, anti-phospho p38 or anti-phospho JNK – Cell Signaling Technology, Beverly, MA) overnight at 4°C. The blots were washed 3 × 5 min with TTBS and incubated for 1 h with HRP-conjugated anti-rabbit IgG Ab 1/2000(Cell Signaling Technology, Beverly, MA). Immunoreactive bands were revealed using a chemiluminescent substrate (ECL, Amersham Biosciences, UK) and chemiluminescence was detected with chemidoc XRS apparatus. The intensity of each blot was measured with the densitometry program Quantity One (Bio-Rad Laboratories, Hercules, CA).

### IL-10 mRNA degradation assay

1 × 10^6 ^HAM/well were stimulated with LPS (1 μg/ml) for 16 h, translation was stopped by using Actinomycin D (2.5 μg/ml) and then, PD98059, SB203580 or medium (25 μM) was added for various time periods. HAM were collected for RNA extraction and real time PCR for IL-10 mRNA was performed.

### MTT assay

2.5 × 10^5 ^HAM/well were incubated with PD98059, SB203580, SP600125 (25 μM) or medium for 24 h. Supernatants were discarded and 100 μl of Thiazolyl Blue Tetrazolium Bromide (MTT, 1 mg/ml) was added to the cells for 1 h. DMSO was then used to stop the reaction and optical density was read at 550 nm.

### Statistical analysis

Comparisons were performed using two-tailed paired t test or Mann-Wittney U test as appropriate. All analyses were performed using a statistics software package (GraphPad Prism, PA, USA). p values < 0.05 were considered as statistically significant.

## Results

### LPS induces IL-10 secretion in human alveolar macrophages

**Figure 1:**
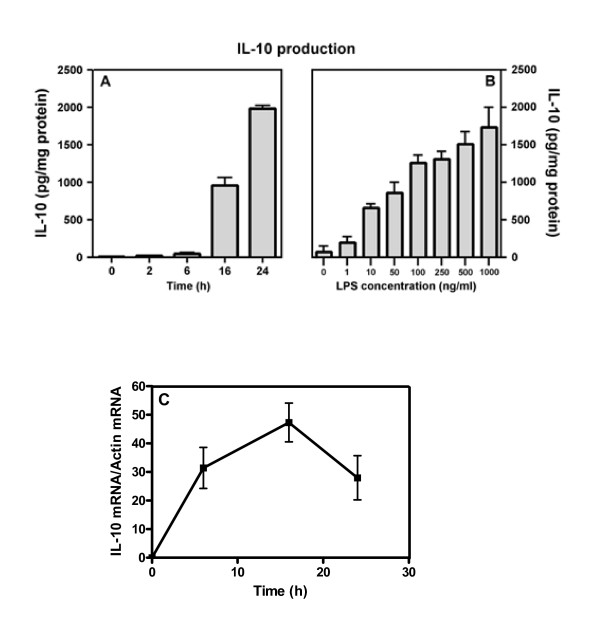
**Time and dose dependency of IL-10 production in HAM stimulated by LPS**. HAM were stimulated with LPS (1 μg/ml) and supernatant were collected after different incubation time and assayed for IL-10 content by ELISA for Panel A and by real time PCR for Panel C. Panel B: HAM were stimulated for 24 hours with increasing concentration of LPS and supernatant were assayed for IL-10 by ELISA.

In a first series of experiments, we evaluated the ability of HAM to release IL-10 after LPS stimulation. Figure 1 panel A shows the production of IL-10 overtime. The release of IL-10 begins after 6 h of incubation and reaches a maximum at 24 h. Moreover, IL-10 production is dose-dependent and linear in the range of LPS concentration between 1 ng/ml and 1 μg/ml (Figure 1, panel B). The time-course of IL-10 induction by LPS was confirmed at gene level by real time PCR (Figure 1, panel C).

To check if IL-10 production is CD14 dependent, we used an anti-CD14 blocking antibody. Preincubation of HAM with a neutralizing anti-CD14 (10 μg/ml) totally inhibits LPS-induced IL-10 (data not shown).

### Activation of MAPkinases by LPS

**Figure 2:**
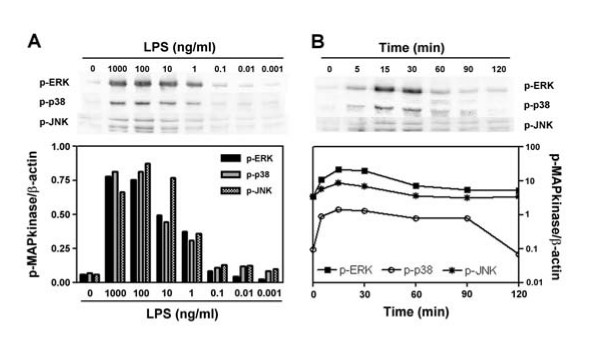
**Activation of MAP kinases in HAM stimulated by LPS**. Panel A: HAM were stimulated 30 min with increasing doses of LPS and phosphorylated ERK, p38 and JNK were detected by Western-blotting as described in Materials and Methods. Panel B: HAM were incubated with LPS (1 μg/ml) during different time and phosphorylated ERK, p38 and JNK were detected by Western-blotting. Below the blots, the graph represents the phosphorylated MAP kinases to β-actin ratio obtained by densitometric analysis of each bands using Quantity One Sotware.

It is well-known that LPS activates ERK, p38 and JNK MAPkinases. The ability of LPS to induce the phosphorylation of ERK, p38 and JNK in human alveolar macrophages was evaluated by western-blotting. As shown on Figure 2, LPS triggers ERK, p38 and JNK phosphorylation in a time- and dose-dependent fashion. Panel A shows that both ERK and p38 MAPkinases reached a maximum of phosporylation at a concentration of 100 ng/ml and are not activated at concentration below 100 pg/ml. Concerning JNK MAPkinase, phosphorylation is dose-dependent in the range of concentration between 0.1 and 10 ng/ml. Experiments of time-dependence show that both ERK and p38 are rapidly phosphorylated (within 5 min) and reached a maximum of activation after 15–30 min followed by a progressive decline and come-back to the basal state after 90 and 120 min for ERK and p38 respectively (panel B). JNK phosphorylation is also rapid (within 5 minutes) and reached its maximum at 15 min but returns to its basal state within 60 min.

### MAPkinases are crucial for IL-10 production

**Figure 3:**
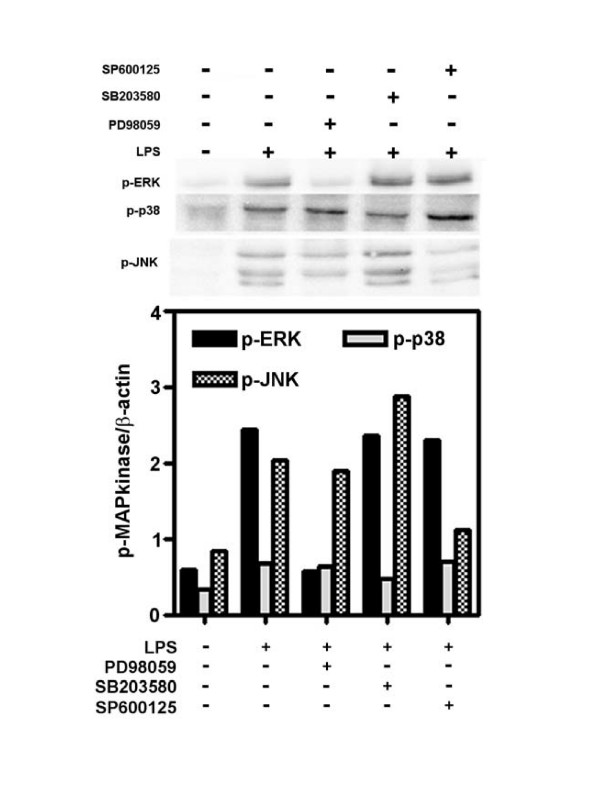
**Inhibition of MAPkinases activation by PD98059, SB203580, SP600125**. HAM were preincubated during 1 h with inhibitors (50 μM) and then stimulated 30 min with LPS (1 μg/ml). Levels of phosphorylated ERK, p38 and JNK MAP kinases were evaluated by Western-blot analysis. Each bands was normalised by performing phospho MAP kinase to β-actin ratio as by the graph representing the densitometric analysis.

**Figure 4:**
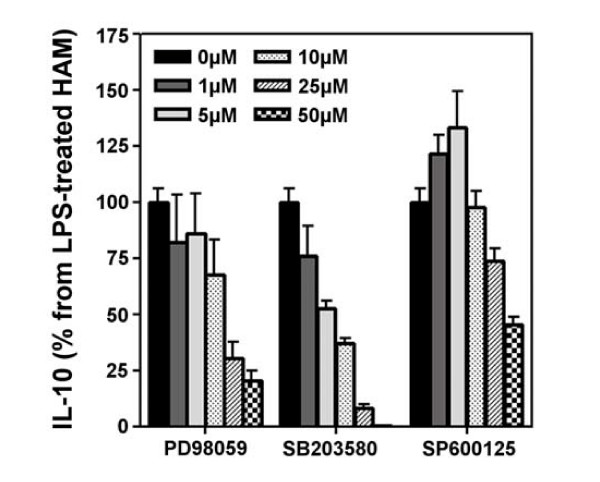
**Effect of specific MAP kinase inhibitors on the production of IL-10 in HAM**. HAM were preincubated (1 h) with increasing concentrations of inhibitors and then were stimulated with LPS (1 μg/ml) during 24 h. Supernatants were assayed to determine the production of IL-10 by ELISA.

Since we have shown that the three MAPkinases were activated following LPS stimulation, we therefore evaluated the precise role of each MAPkinase in the production of IL-10. To this aim, PD98059, SB203580 and SP600125, three specific inhibitors of ERK, p38 and JNK respectively were used. First, the specificity of each inhibitor was checked by western-blot (Figure 3) and cell toxicity was assessed by the MTT assay (Table 1). No significant cytotoxicity was observed for the three inhibitors at 25 μM while 50 μM of SB or SP induced a 30–35% reduction in MTT reduction (Table 1). Pre-treatment of HAM with PD98059 (50 μM) clearly inhibits LPS-induced ERK activation and was without significant effect on p38 and JNK phosphorylation (Figure 3). SB203580 (25 μM) partially inhibits LPS-induced p38 phosphorylation without affecting ERK and JNK activation while SP600125 (50 μM) is able to prevent the phosphorylation of JNK MAPkinase after LPS stimulation without affecting ERK phosphorylation. In the latter conditions, phosphorylation of p38 MAPkinase is slightly increased. Having shown that these three inhibitors were specific of each MAPkinase, we used them to evaluate the involvment of MAPkinases in the production of IL-10. As shown on Figure 4, PD98059, SB203580 and SP600125 dose-dependently inhibit LPS-induced IL-10 in HAM. At the maximum concentration (50 μM), SB203580 totally inhibits IL-10 production (more than 99% of inhibition) whereas PD98059 is slightly less active (80% of inhibition). The specific inhibitor of JNK MAPkinase, SP600125, at its maximal concentration, only reduced IL-10 production by 50%.

**Table 1:** MTT-based cytotoxicity assay of MAPk inhibitors in HAM. HAM were incubated for 24 h with PD98059, SB203580, SP600125 or medium at different concentrations, and cell viability was assessed by using the MTT reduction assay, as described in Methods. Results are mean ± SD of one representative experiment in triplicates (expressed as % of control); *p value < 0.05 (one sample t-test).

**Concentration of inhibitors**	**PD98059**	**SB203580**	**SP600125**
**1 μM**	96.2 ± 20.26	92.7 ± 14.2	92.6 ± 19.5
**5 μM**	93.4 ± 14	85.3 ± 34.6	104.9 ± 33.5
**10 μM**	79.9 ± 14.2	104.1 ± 15.7	110.1 ± 11
**25 μM**	122.3 ± 6.9	82.8 ± 7.2	89.3 ± 9
**50 μM**	104.6 ± 20.6	66.2 ± 10.1*	65.2 ± 9*

### Role of Sp1 transcription factor in the production of IL-10

**Figure 5:**
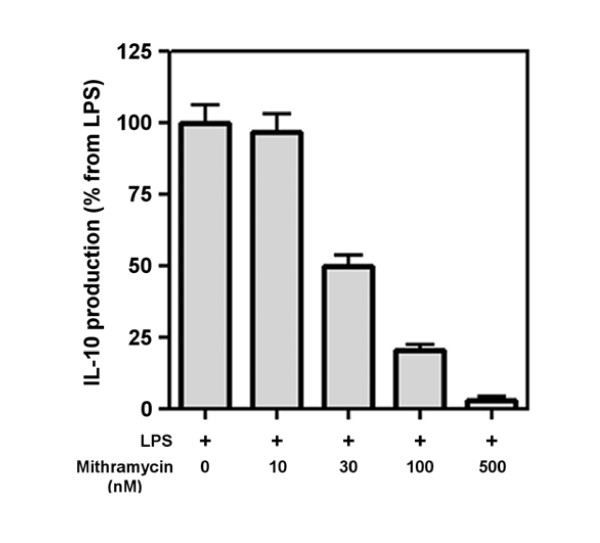
**Effect of mithramycin on IL-10 production in HAM**. HAM were preincubated (1 h) with increasing concentration of mithramycin and were stimulated with LPS (1 μg/ml) during 24 h. Supernantants were assayed by ELISA to determine IL-10 production.

**Figure 6:**
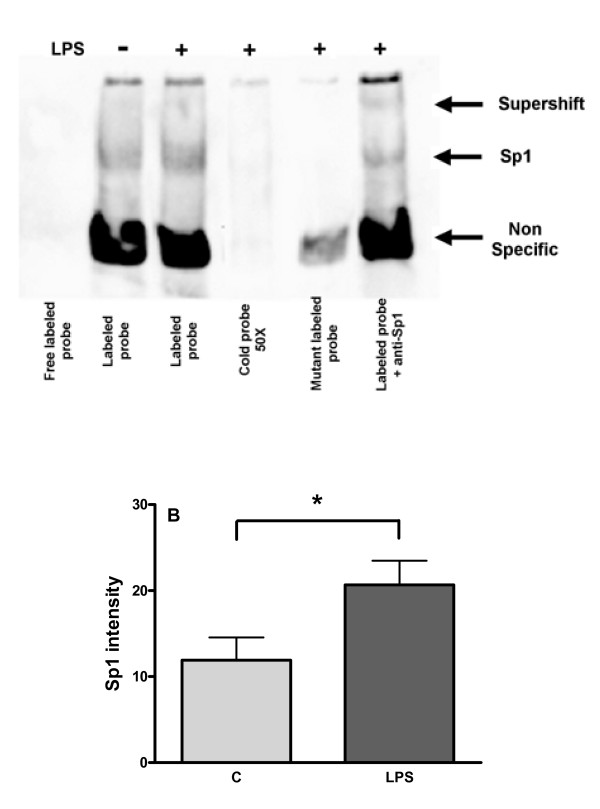
**EMSA analysis of Sp1 binding activity in nuclear proteins of HAM**. Panel A : HAM were treated or not with LPS (1 μg/ml) during 2 h. Nuclear proteins were extracted following procedure described in Materials and Methods. Lane 1: free labelled probe only (without nuclear proteins) – Lane 2: Nuclear proteins from control HAM incubated with labelled probe specific for Sp1 – Lane 3: idem lane2 except that HAM were treated 2 h with LPS – Lane 4: idem lane 3 except that an excess of unlabelled (cold) probe was added in the binding reaction – Lane 5: idem lane 3 except that a mutant labelled probe (mutation in consensus binding site) is used instead of the specific labelled probe for Sp1 – Lane 6: idem lane 3 except that nuclear proteins were preincubated with a specific anti-Sp1 antibody before addition of the specific labelled probe. Panel B represents a quantitative analysis of a EMSA gel from HAM following stimulation by LPS. Data are mean ± SD from 6 experiments; *p value = 0.0411 (Mann-Whitney *U *test).

Sp1 transcription factor is one of the main transcription factor regulating IL-10 transcription in monocytes/macrophages. In order to evaluate the involvement of Sp1 in IL-10 production in HAM, we first used mithramycin as a specific inhibitor of Sp1. As shown on Figure 5, mithramycin dose-dependently inhibits LPS-induced IL-10 production in HAM with a maximal inhibition at 500 nM. Secondly, we set up an EMSA assay to confirm that Sp1 was activated and that the inhibition observed with mithramycin was due to an effect on Sp1. Activation of Sp1 following LPS stimulation was assessed using a specific probe containing the consensus binding site for Sp1. Figure 6 represents a representative EMSA gel where HAM have been activated by LPS during 2 h. As control, the free probe (without nuclear extract protein) shows no band whereas in the control and LPS-treated HAM, two bands (one major intense and one light) are found in both conditions whereas the light band is more present in LPS-treated HAM. To determine which of these bands is specific to Sp1, the following controls have been performed. First, an excess of cold probe totally switch off all the bands. Secondly, the use of a mutant probe (mutation in the consensus binding site of Sp1) gives only one band corresponding to the major band meaning that this band is a non-specific one. Thirdly, addition of an anti-Sp1 antibody decreased the light band and induces a super-shift. Therefore, we can conclude that the light band represents the complex Sp1/probe.

**Figure 7:**
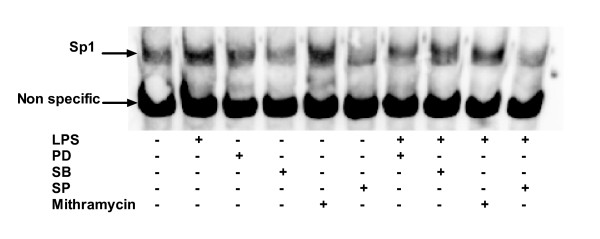
**Sp1 binding activity in nuclear proteins of HAM**. HAM were preincubated 1 h or not with inhibitors (50 μM for PD98059, SB203580 and SP600125 or 500 nM for mithramycin) and then stimulated with LPS (1 μg/ml) during 2 hours. Control HAM were HAM cultured with medium alone. Nuclear proteins were extracted as previously described and were then incubated in the binding buffer with specific labelled probe and then subjected to electrophoresis for separation of the complexes.

Preliminary experiments have shown that maximum Sp1 activation following LPS stimulation is reached between 1 and 2 h (data not shown), and that LPS was up-regulating nuclear translocation of Sp1 (Figure 6B). In the following experiments, we have assessed the role of the three MAPkinases using their specific inhibitors in the activation of Sp1. Figure 7 shows that PD98059, SB203580 and SP600125 alone did not influence the binding of Sp1 to the probe. However, in LPS-treated HAM, these three inhibitors decreased the activation of Sp1 induced by LPS.

### Effects of MAPkinases and Sp1 inhibitors on IL-10 mRNA

**Figure 8:**
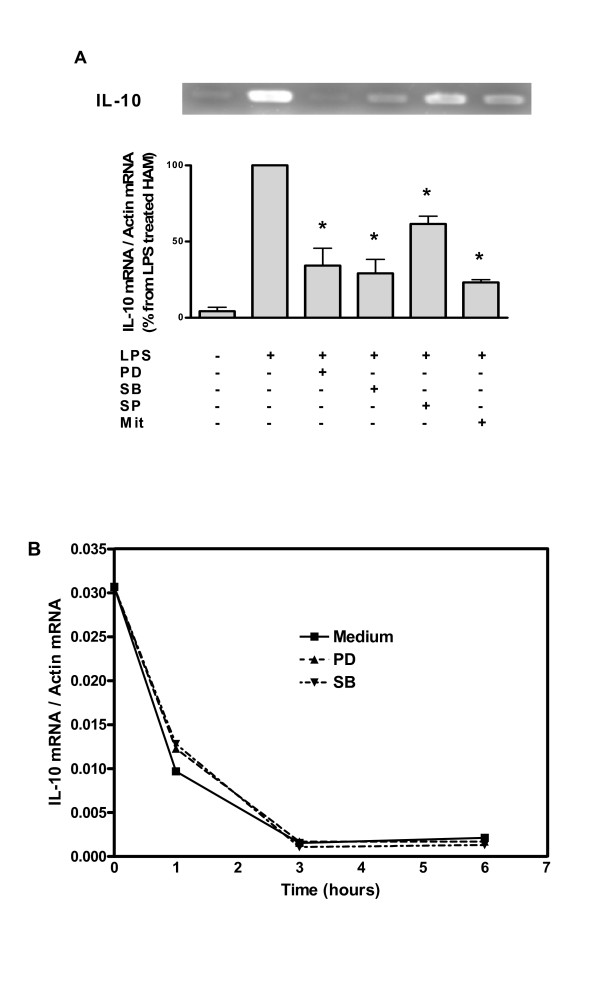
**IL-10 mRNA in HAM stimulated with LPS**. Panel A : HAM were preincubated 1 h with inhibitors (25 μM for PD98059, SB203580 and SP600125 or 100 nM for mithramycin) and then stimulated for 24 h with LPS. RNA was extracted as described in details in Materials and Methods. After reverse transcription, specific primers were used to amplify fragment of IL-10 mRNA and the relative abundance of IL-10 compared to β-actin is represented in the graph. Data are mean ± SD from 3 experiments; *p value < 0.05 versus (One sample t test). Panel B: HAM were incubated with LPS (1 μg/ml) for 16 h, translation was stopped using Actinomycin D 2.5 μg/ml and inhibitors (PD98059 or SB203580, 25 μM) were added. IL-10 mRNA was assayed at different time points by real time PCR and corrected for β-actin mRNA.

To further demonstrate the role of the MAPkinases and the Sp1 transcription factor in the production of IL-10, we performed quantitative assay of IL-10 mRNA using real-time PCR. This technique allows the precise quantification of mRNA using specific primers. Figure 8 shows the relative quantification of IL-10 mRNA after different treatments. First, as positive control, LPS induced IL-10 mRNA production in HAM (Figure 8, panel A). Treatment of HAM with PD98059 (50 μM), SB203580 (50 μM), SP600125 (50 μM) or mithramycin (100 nM), decreased IL-10 mRNA induced by LPS. This inhibition is more pronounced for PD98059 (>99% inhibition) and SB203580 (90% inhibition) inhibitors whereas mithramycin (75% inhibition) and SP600125 (45% inhibition) are less active.

To assess a potential effect of PD and SB inhibitors on post-transcriptional mechanisms of regulation for IL-10 production, real time PCR for IL-10 mRNA was performed in HAM treated with Actinomycin D. Figure 8, panel B shows that both MAPK inhibitors did not affect significantly IL-10 mRNA degradation following LPS stimulation.

## Discussion

Lung homeostasis relies on the equilibrium between the induction of efficient innate defensive responses to inhaled infectious microorganisms and equally effective mechanisms to downregulate the inflammatory response to initiate resolution and tissue repair. As a predominant immune effector cell in the airspaces, the alveolar macrophage is critical to these defence processes. Thus they produce a vast array of cytokines and inflammatory mediators in particular after LPS stimulation, including TNF-α and IL-10 as prototypic pro-inflammatory and anti-inflammatory cytokines, respectively. While the signaling events that mediate TNF-α in HAM have been extensively studied [20-23,19], those responsible for IL-10 production have not been well characterized.

The present study is the first one showing that ERK, p38, JNK and Sp1 are involved and essential in LPS-induced IL-10 expression in HAM. These factors act at the transcriptional level, as IL-10 mRNA stability was not affected by MAPK inhibitors. It is well known that LPS drives intracellular signaling pathways such as MAPKs and NF-κB [23,24] to activate several pro-inflammatory genes including cyclooxygenase-2 [25], inducible nitric oxygen synthase [26], TNF-α and IL-1β [27]. In the present study, we found that in HAM the MAPK signaling pathways were also involved in LPS-induced gene activation of IL-10, a major anti-inflammatory factor. A recent study in murine macrophage raw 264.7 cells also reported that MAPKs were necessary in IL-10 expression by LPS [28] whereas other studies showed that only MAPK p38 was essential for IL-10 expression induced by LPS and other ligands [29-33]. Our study not only showed that all MAPKs were necessary but that they also played an important role in the downstream activation of Sp1 trancription factor.

Sp1 is the founding member of a family of zinc finger transcription factors, which includes at least four Sp transcription factors [34,35]. Among the Sp transcription factors, Sp1 has been extensively studied [36] and is known to be widely expressed and to play a role in the regulation of a vast array of genes. Thus, while not excluding a role for other nuclear factors our data confirm previous studies showing that IL-10 gene expression is controlled by the transcription factor Sp1 [13,14,37] since mithramycin, an inhibitor of Sp1, almost completely abrogated LPS-induced IL-10 production at the mRNA and protein level. EMSA assay confirmed this observation as it showed that mithramycin decreased the activation of the nuclear protein Sp1 after LPS stimulation. Transfection of HAM by antisense oligonucleotides to Sp1 could not be used as another approach to confirm the role of Sp1 in IL-10 induction by LPS, as after the time period required to silence Sp1 expression (minimum 16–20 hrs) following 4 hrs-transfection, HAMs were not anymore responsive to LPS for IL-10 production (data not shown). Interestingly, inhibition of MAPKs by their specific inhibitors, also abolished LPS-induced Sp1 activation. These results are in accordance with previous studies [14,38-42] showing that Sp1 phosphorylation can be induced by ERK and p38 MAP kinases. In addition the present study also showed that JNK MAP kinase is also required for the activation of Sp1 induced by LPS. The three MAP kinases seem however to have different contributions to LPS-induced IL-10 in HAM, with a prominent role of p38 and ERK.

IL-10 production by alveolar macrophages has been debated since some authors described the inability of alveolar macrophages to produce IL-10 [43-45]. Other investigators related this to a reduced production of IL-10 to allergic inflammation [46]. Our data clearly confirm IL-10 production by normal HAM [47-54], provide new information on the mechanisms involved in this production and complete the studies of Boehringer et al [18] who has studied the role of PP1 and PP2A in the regulation of LPS-induced IL-10 in HAM.

## Conclusion

Our study demonstrates the contribution of MAP kinases to IL-10 expression in HAM upon endotoxin activation, indicating that ERK and p38 and to a lesser extent JNK are involved. In addition we show that ERK, p38 and JNK are able to trigger the phosphorylation of Sp1, the major transcription factor for the IL-10 gene. These findings are highly relevant to lung immunity, alveolar macrophages assuming front-line defense mechanisms and IL-10 representing a key factor for mucosal tolerance and resolution of inflammatory responses.

## Competing interests

The author(s) declare that they have no competing interests.

## Authors' contributions

HC supervised all the experiments, performed the data analysis and wrote the manuscript. AG carried out the experiments. CP was involved in the design of the study and in writing of the manuscript. YS was involved in the design of the study, writing of the manuscript and interpretation of the data. All authors read and approved the final manuscript.
